# Aortic Pseudoaneurysm in an Unusual Site: Difficult Diagnosis by Echocardiography

**Published:** 2019-04

**Authors:** Ali Hosseinsabet, Hasan Aghagani, Khalil Forozannia, Ahmad Yaminisaharif

**Affiliations:** *Tehran Heart Center, Tehran University of Medical Sciences, Tehran, Iran.*

**Keywords:** *Aorta*, *Aneurysm, false*, *Echocardiography*, *Diagnosis*

A 54-year-old woman was admitted to our hospital with a fever of 1 week’s duration and a distal embolic event 4 days previously in the second and third digits of her right hand. She had a history of aortic valve replacement (STj#19) 2 months earlier as well as mitral valve replacement (STj#26) and tricuspid valve repair 10 years before. Lab data showed an increased white blood cell count and an elevated erythrocyte sedimentation rate. Blood culture was positive only in 1 round with *Pseudomonas aeruginosa* growth, which was not compatible with the patient’s good general condition. Transthoracic and transesophageal echocardiography, abdominal sonography, and computed tomography (CT) scan of the right hand were unremarkable. The patient was treated for infectious endocarditis with antibiotics for 6 weeks and then discharged. On follow-up transthoracic echocardiography, 1 month after discharge, an echo-free space in the posterolateral wall of the ascending aorta was detected. The second transesophageal echocardiography was highly suggestive of a pseudoaneurysm in the ascending aorta (Figure 1A & Video 1), which was subsequently confirmed by a CT angiography of the ascending aorta (Figure 2). For a better evaluation, aortography was done and it revealed a pseudoaneurysm in the posterolateral wall of the ascending aorta (Figure 3 & Video 2). A review of the previous transthoracic and transesophageal echocardiography images demonstrated that this pseudoaneurysm had been missed because it was filled with a thrombosis (Figure 1B & Video 3). The ascending aorta was normal in aortography before the second surgical operation. It appears that the most probable scenario was thrombosis formation in an iatrogenic aortic pseudoaneurysm at an unusual site, causing such presentations. The thrombosis was resolved with meticulous anticoagulation, leading to the clarification of the pseudoaneurysm. The administration of an anticoagulant was obligatory in this patient because of the presence of 2 mechanical valves. Nevertheless, this administration could lead to catastrophic events such as a ruptured ascending aorta, so the early detection of this complication may reduce the risk to the patient and confer an appropriate treatment. We repaired our patient’s pseudoaneurysm via surgery, and she was discharged in good physical condition. The development of an aortic pseudoaneurysm is an uncommon complication of open-heart surgery, and it usually forms in the anterior wall; nonetheless, its formation in the posterior wall is rare and can be due to the deep cannulation of the ascending aorta during surgery. The presence of this complication should, therefore, be kept in mind by any cardiologist who encounters a patient with an embolic event and previous cardiac surgery.

**Figure 1:**
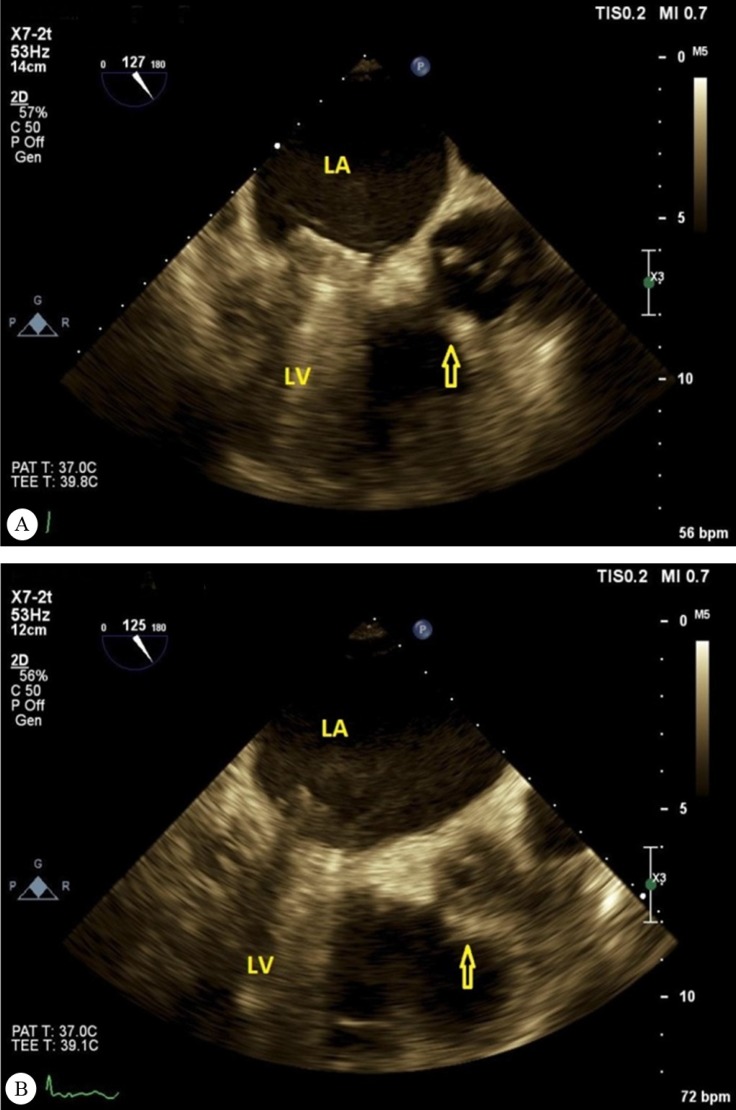
A) Pseudoaneurysm (arrow) of the ascending aorta in the second transesophageal echocardiography (long-axis view), depicted after the resolution of the thrombosis. B) The same pseudoaneurysm (arrow) of the ascending aorta in the same view in the primary transesophageal echocardiography, presenting a challenge in diagnosis because it was filled with a thrombosis.

LA, Left atrium; LV, Left ventricle

**Figure 2:**
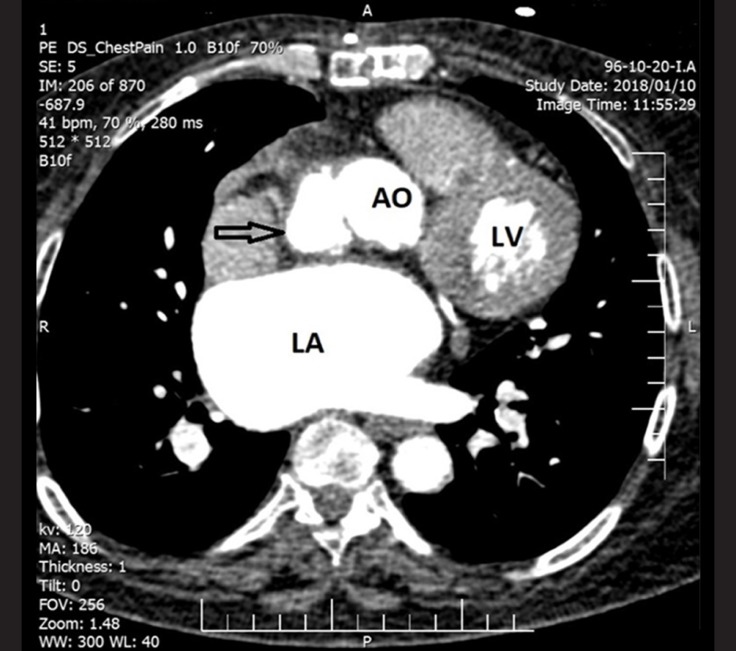
Computed tomography angiography of the aorta in a cross-sectional slice, showing the pseudoaneurysm (arrow) in the posterolateral wall of the ascending aorta

AO, Ascending aorta; LA, Left atrium; LV, Left ventricle

**Figure 3:**
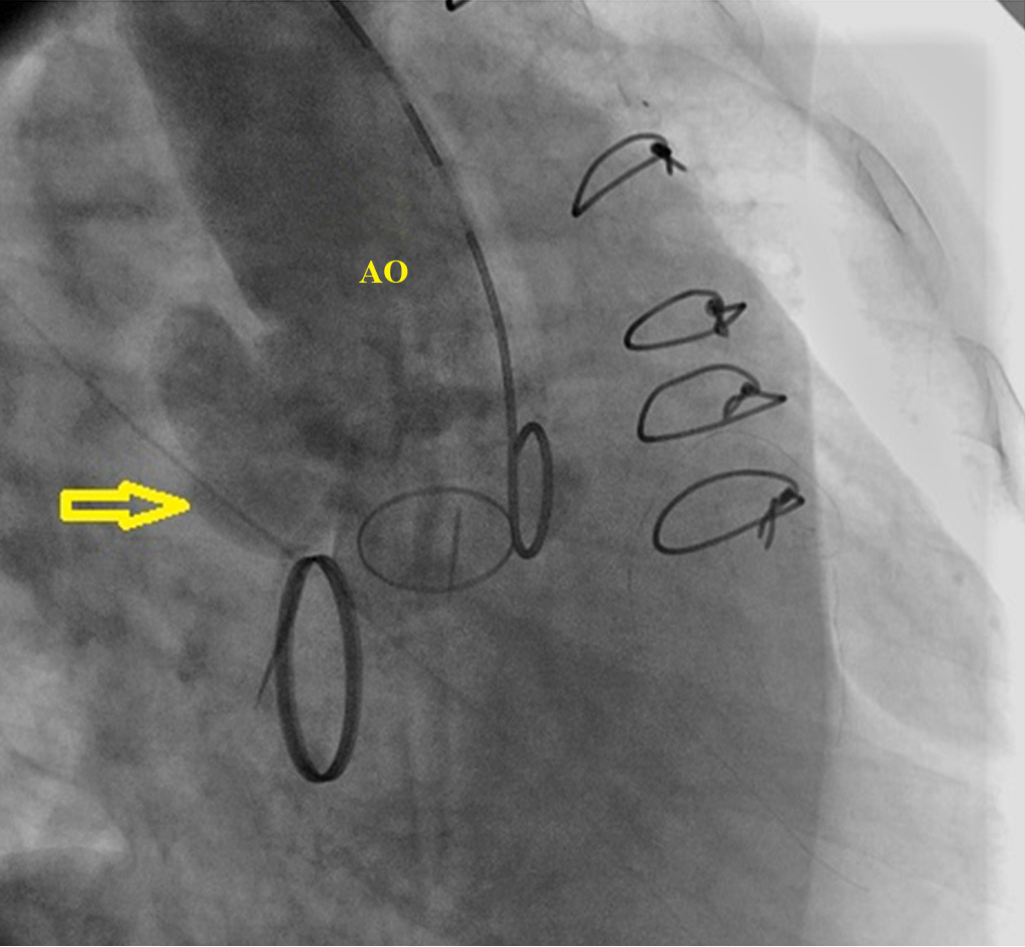
Aortography of the ascending aorta in the right anterior oblique view, showing the pseudoaneurysm (arrow)

## Notes:

***This paper should be cited as: ***Hosseinsabet A, Aghagani H, Forozannia K, Yaminisaharif A. Aortic Pseudoaneurysm in an Unusual Site: Difficult Diagnosis by Echocardiography. J Teh Univ Heart Ctr 2019;14(2):92-93.

## Notes:

***To watch the following videos, please refer to the relevant URLs: ***

http://jthc.tums.ac.ir/index.php/jthc/article/view/904/841

Video 1. Pseudoaneurysm of the ascending aorta is depicted in the long axis-view of the ascending aorta in transesophageal echocardiography after the resolution of the thrombosis

http://jthc.tums.ac.ir/index.php/jthc/article/view/904/842

Video 2. Aortography of the ascending aorta, showing the pseudoaneurysm

http://jthc.tums.ac.ir/index.php/jthc/article/view/904/843

Video 3. Pseudoaneurysm of the ascending aorta, which was missed by transesophageal echocardiography (long-axis view) of the ascending aorta because it was filled with a thrombosis

